# Simulation of leaf curl disease dynamics in chili for strategic management options

**DOI:** 10.1038/s41598-020-79937-0

**Published:** 2021-01-13

**Authors:** Buddhadeb Roy, Shailja Dubey, Amalendu Ghosh, Shalu Misra Shukla, Bikash Mandal, Parimal Sinha

**Affiliations:** Division of Plant Pathology, ICAR-Indian Agricultural Research Institute, New Delhi, 110012 India

**Keywords:** Microbiology, Plant sciences

## Abstract

Leaf curl, a whitefly-borne begomovirus disease, is the cause of frequent epidemic in chili. In the present study, transmission parameters involved in tripartite interaction are estimated to simulate disease dynamics in a population dynamics model framework. Epidemic is characterized by a rapid conversion rate of healthy host population into infectious type. Infection rate as basic reproduction number, *R*_0_ = 13.54, has indicated a high rate of virus transmission. Equilibrium population of infectious host and viruliferous vector are observed to be sensitive to the immigration parameter. A small increase in immigration rate of viruliferous vector increased the population of both infectious host and viruliferous vector. Migrant viruliferous vectors, acquisition, and transmission rates as major parameters in the model indicate leaf curl epidemic is predominantly a vector -mediated process. Based on underlying principles of temperature influence on vector population abundance and transmission parameters, spatio-temporal pattern of disease risk predicted is noted to correspond with leaf curl distribution pattern in India. Temperature in the range of 15–35 °C plays an important role in epidemic as both vector population and virus transmission are influenced by temperature. Assessment of leaf curl dynamics would be a useful guide to crop planning and evolution of efficient management strategies.

## Introduction

Begomoviruses (family *Geminiviridae*) are the most destructive plant viruses that cause diseases like leaf curl, mosaic, yellow mosaic and yellow vein mosaic in numerous crop plants in the tropical and subtropical world. Leaf curl disease in chili is known to cause by several begomoviruses of which Chili leaf curl virus (ChiLCV) is the most predominant in India^1–3^. ChiLCV is efficiently spread by whitefly (*Bemisia tabaci*) which is abundant year-round in tropical and subtropical climates where wide variety of hosts serves as reservoirs^3–7^. Leaf curl in chili (ChiLCD) is a common disease but recent frequent epidemic outbreaks witnessed repeatedly in the chili growing areas of Central and Southern India is now a growing concern^3,8–10^. ChiLCD is the most damaging in terms of yield loss across the cropping regions^11^. In severe cases, even up to 100% losses of marketable fruit have been reported^12^. In absence of epidemiological information so far no definite management strategy has been evolved. Interaction or contact rate between viruliferous vector and the growing host at initial stages is crucial in epidemic development and thus the period of high contact rate appears critical point of interventions^13,14^. Simulation of epidemics resulted from complex host–vector interaction is of great interest for on-farm management and assessment of agricultural policies and practices^15–17^. Time of whitefly population abundance, especially the growth of viruliferous flies and its effect on host associated leaf curl risk at regional and global scales is of great interest for precise interventions. Type of intervention that ensures low contact rates and keeps the proportion of infectious host at minimum level, are useful in designing precise management strategies^18^. Chili being cultivated in a wide variety of agro-ecosystems in India, disease dynamics in relation to tripartite interaction and transmission behavior are useful for evolving management strategies universally applicable to all climates. Understanding the complex host–vector interactions is also necessary to assess emerging disease risk under rapidly changing cropping patterns as well as global temperature rise scenario^6,19,20^.

Leaf curl epidemic is resulted from a complex tripartite interaction between host, vector, and virus where virus transmission plays a central role^15^. Population dynamics models have been used to characterize epidemics resulted from tripartite interaction especially for whitefly transmitted begomovirus in cassava^21–24^ and tomato^25^. Environmental factor particularly temperature plays important role in transmission and vector movements^17,22,26^. Virus transmission, latent period, infectious period, replication, titer, movements and symptoms expression etc. are directly linked to temperature^27–29^. For prediction of whitefly population abundance, temperature-dependent model, explaining developmental rates of each life stage of *B. tabaci*, is used^30–33^. Availability of large scale geo-spatial temperature data facilitates prediction of spatio-temporal distribution of whitefly abundance. To unfold tripartite interaction in the agroecosystem, it is necessary to link the time of whitefly abundance in relation to virus transmission and actual disease risk.

Establishment of disease dynamics in terms of epidemic parameters such as transmission and acquisition rate, prediction of vector abundance time and associated disease risk period may help to develop a testing framework for evolving precise intervention strategies^15,34^. Further, assessment of temperature influence on transmission parameters and prediction of potential geographical distribution in terms of whitefly abundance is likely to shed light on tripartite interaction which may provide insights for developing a rational management policy.

The current paper has addressed the estimation of transmission parameters for simulation of leaf curl dynamics and explaining tripartite interaction in a population dynamics modeling framework. Leaf curl disease dynamics established was used to identify the most sensitive parameter contributing to the growth of infectious host and viruliferous whitefly population in the field. Temperature influence on transmission parameters and whitefly population abundance was used for approximation of disease risk and compare known leaf curl disease distribution patterns in the chili growing areas of the subcontinent.

## Results

### Leaf curl virus (ChiLCV) transmission in chili

**Figure 1:**
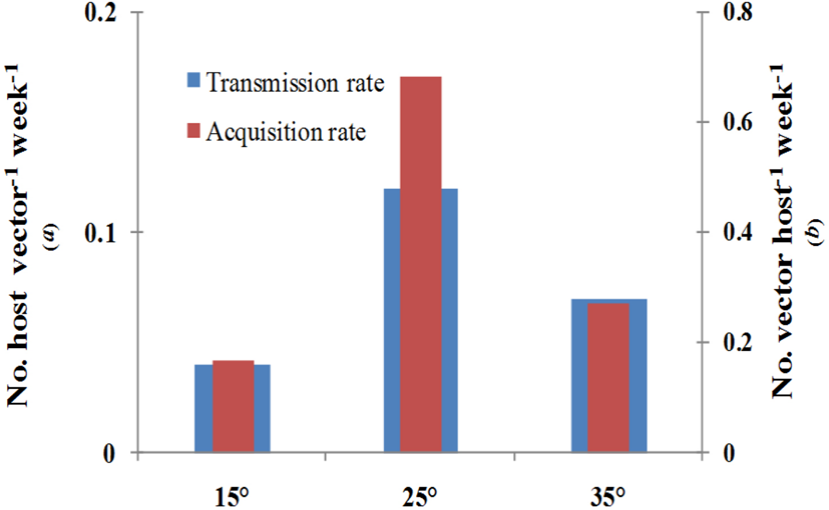
Transmission parameter estimates at 15 °C, 25 °C and 35 °C on susceptible chili cultivar (cvHPH-1041) under semi-controlled conditions (eight plants grown in insect-proof cage; ten viruliferous whiteflies released through suction device; 7 days after release flies were killed spraying Confidor (@ 1 ml/L), blue bar = transmission rate (*a)* and red bar = acquisition rate (*b*); *a* and *b* estimated fitting the equation *dS/dt* = *– aVS* and *dV/dt* = *b XI* = *b(1 − V)I*.

Transmission parameters, estimated under semi-controlled condition, were observed to vary in response to temperature**.** Transmission or inoculation rate as the number of plant/vector/week was 0.12 at 25 °C as compared to 0.04 and 0.07 at 15 °C and 35 °C, respectively (Fig. 1). The number of vectors used in the inoculation experiment influenced the transmission rate. The transmission rate was nine times higher when plants were inoculated with one viruliferous vector as compared to ten. Therefore, the transmission rate was affected by vector aggregation as well as temperature. Acquisition rate at 25 °C was observed to be 0.70 vector/plant/week which was higher than that at 15 °C and 35 °C where the values were in the range of 0.17 to 0.28. Within 20 min, vectors were observed to acquire virus and by 6 h of feeding access period, almost all the vectors were found to acquire ChiLCV as judged by the positive result in PCR test. Temperature response was typically follow a unimodal pattern as the transmission parameter values were observed to increase with the increase in temperature up to a favorable point. Higher transmission and acquisition efficiency at moderate temperature (25 °C) than at lower (15 °C) and decreased afterword higher ranges of temperatures (35 °C) indicated transmission parameters are governed by optimal and suboptimal ranges of temperature.

It appeared leaf curl disease or incidence distribution would likely be affected by prevalent temperature in time and location as virus transmission is influenced by temperature.

### Leaf curl incidence as proportion of leaf curl infection and virulifreous vector in chili field

In the experimental field, chili plants were noted to have an infection from the very first week of transplantation as a proportion (0.01) of samples tested PCR-positive for ChiLCV (Table 1). Proportion of infection was observed to increase rapidly to 0.52 within the fifth week period and by 10th week reached 0.76 level. Population of viruliferous whitefly (V) from the time of transplantation was 0.65 and went on increasing and reached 0.98 by the end of 12th week. Whitefly population sampled from the field (brinjal, cucurbits, tomato and *Leucana leucocephala*) was observed to be PCR-positive even at the time of transplantation and proportion of viruliferous vector was estimated to be 0.65.

**Table 1:** Proportion (*P*) of infectedchilli plant (*I*) and viruliferous whitefly (*V*) population tested PCR-positive^a^ at IARI-New Delhi experimental field, on cvHPH-1041 (Syngenta), transplanted in 2019.

Date	Crop growth	Julian week	Proportion of leaves/whitefly PCR-positive (P*)	
			Infected plant (*I*)	Viruliferous whitefly (*V*)
1.6.19	Sowing of seedling	33	0	
		34	0	
12.6.19		35	0	
		36	0	0.65
13.8.19	Transplanting	37	0	0.65
		38	0.01	0.66
2.9.19	Leaf curl symptoms observed	39	0.02	0.67
		40	0.08	0.67
10.10.19	Leaf curl incidence progress	41	0.12	0.73
		42	0.34	0.83
		43	0.38	0.87
		44	0.40	0.88
11.11.19	Majority of plants in reduced size due to leaf curl	45	0.52	0.89
		46	0.61	0.90
		47	0.65	0.91
		48	0.72	0.92
		49	0.75	0.93
		50	0.76	0.95
		51	0.80	0.96
28.12.19	Terminated	52	0.81	0.98

^a^Chili leaves or whiteflies were tested by PCR with universal primers to ChiLCV.

*P = *1 − ((n* − *X)/n)*^*1/m*^ where *n* = number of groups for the leaves or whiteflies; *m* = number of leaves or whiteflies in each group; *X* = number of groups tested PCR- positive.

It appeared that chili planting during August was at very high-risk period when a high proportion of viruliferous vector already existed nearby field or in border plants.

### Population dynamics model for simulation of leaf curl epidemic in chili

**Figure 2:**
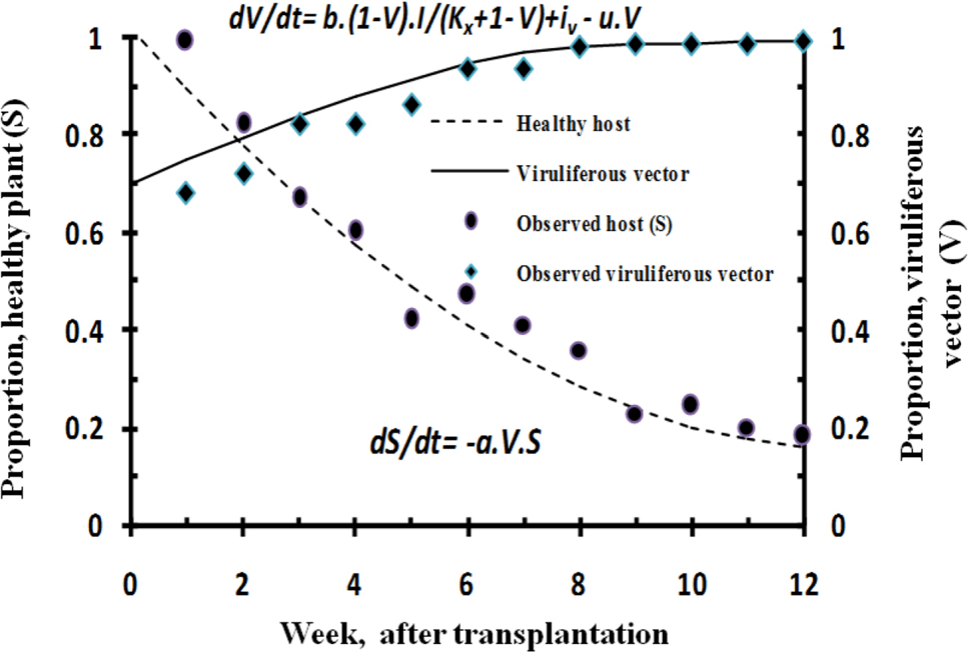
Simulated dynamics of healthy host (*S*) and viruliferous whitefly population (*V*) on susceptible chili cultivar(cvHPH-1041) grown (August–December, 2019) under natural infection conditions at experimental field, New Delhi; Model fitting parameters; transmission rate *a* = 0.12; acquisition rate *b* = 0.70; immigration rate (*i*_*v*_) = 0.34 and; mortality rate = 0.34; K_x_ = 2.8; RMSE = 0.0105 (host) and 0.0167 (vector).

For simulation of epidemics, population dynamics models were fitted on leaf curl incidence data observed under field conditions. Transmission parameters estimated under semi-controlled conditions were tuned to match population of healthy host and viruliferous vector. Values of model parameters and variables tuned or adjusted for leaf curl epidemic analysis are given in Table 2. Population of healthy host (*S*) was predicted fitting host dynamics model (*dS/dt* = − *aVS*) and found to correspond with the observed proportion of healthy chili plants in the field (Fig. 2). Transmission or inoculation rate (*a* = 0.12) was approximately close to simulate healthy host population in the field as indicated by low RMSE. Acquisition rate (*b* = 0.70) estimated under semi-controlled conditions and used to simulate virulifeorus vector population fitting vector population dynamics model *(dV/dt* = *b[1* − *V]I/(K*_*x*_ + *1* − *V)* + *i*_*v*_ − *e*_*v*_ − *uV)*was in good agreement as indicated by the low RMSE value. Immigration rate (*i*_*v*_) was approximated to be 0.34 flies plant^−1^ week^−1^ with high infectivity (0.65) which led to a high infection rate in chili within 2-month period of transplantation. Adjustment of death rate to 0.34 indicated high mortality than the usual rate. Dynamics of vector population were observed to be better explained by the Michaelis–Menten parameter (*K*_*x* =_ *2.8*) than the simple kinetic parameter.

**Table 2:** Model parameters and variables used for leaf curl epidemic analysis in chili.

Symbol	Name	Estimated range/adjusted
*a*	Transmission rate at 25 °C	0.10–0.90 plant vector^−1^ week^−1^
*K*_*x*_	Michaelis–Menten for rate of change in healthy vector (X)	2.8 healthy vector week^−1^
*b*	Acquisition rate at 25 °C	0.70 vector plant week^−1^
*i*_*v*_	Immigration rate for viruliferous vector	0.34 vector week^−1^
*e*_*v*_	Emigration rate for viruliferous vector	0.005 vector week^−1^
*i*_*x*_	Immigration rate for Aviruliferous vector	0.05 vector week^−1^
*e*_*x*_	Emigration rate of aviruliferous vector	0.005 vector week^−1^
*u*	Death rate for aviruliferous as well as viruliferous vector	0.34 vector week^−1^
*c*	Birth rate for aviruliferous as well as viruliferous vector	0.05 vector week^−1^
*S*	Healthy plant (1 − I)	0–1.0
*I*	Infectious plant (PCR-positive)	0–0.99
*V*	Viruliferous vector (PCR-positive)	0.65–0.97
S*	Healthy plant (1 − I*) at equilibrium	0–1.0
X*	Aviruliferous vector (1 − V)	0–1.0
V*	Viruliferous vector population at equilibrium	0–1.0
I*	Infectious population at equilibrium	0–1.0

Estimation of basic reproduction number (R_0_) indicated from one infected host about 13.54 plants are infected in the next generation. High transmission rate resulted in quick growth in the leaf curl epidemic.

Fitted population dynamics model on field data based on transmission parameters (*a* and *b* estimated) appeared to represent the dynamic tripartite interaction in the field. Thus tripartite interaction captured in the model is likely to represent the dynamics of host and viruliferous vector population that were involved in the epidemic process.

### Critical epidemic parameters in leaf curl disease dynamics-sensitivity analysis

**Figure 3:**
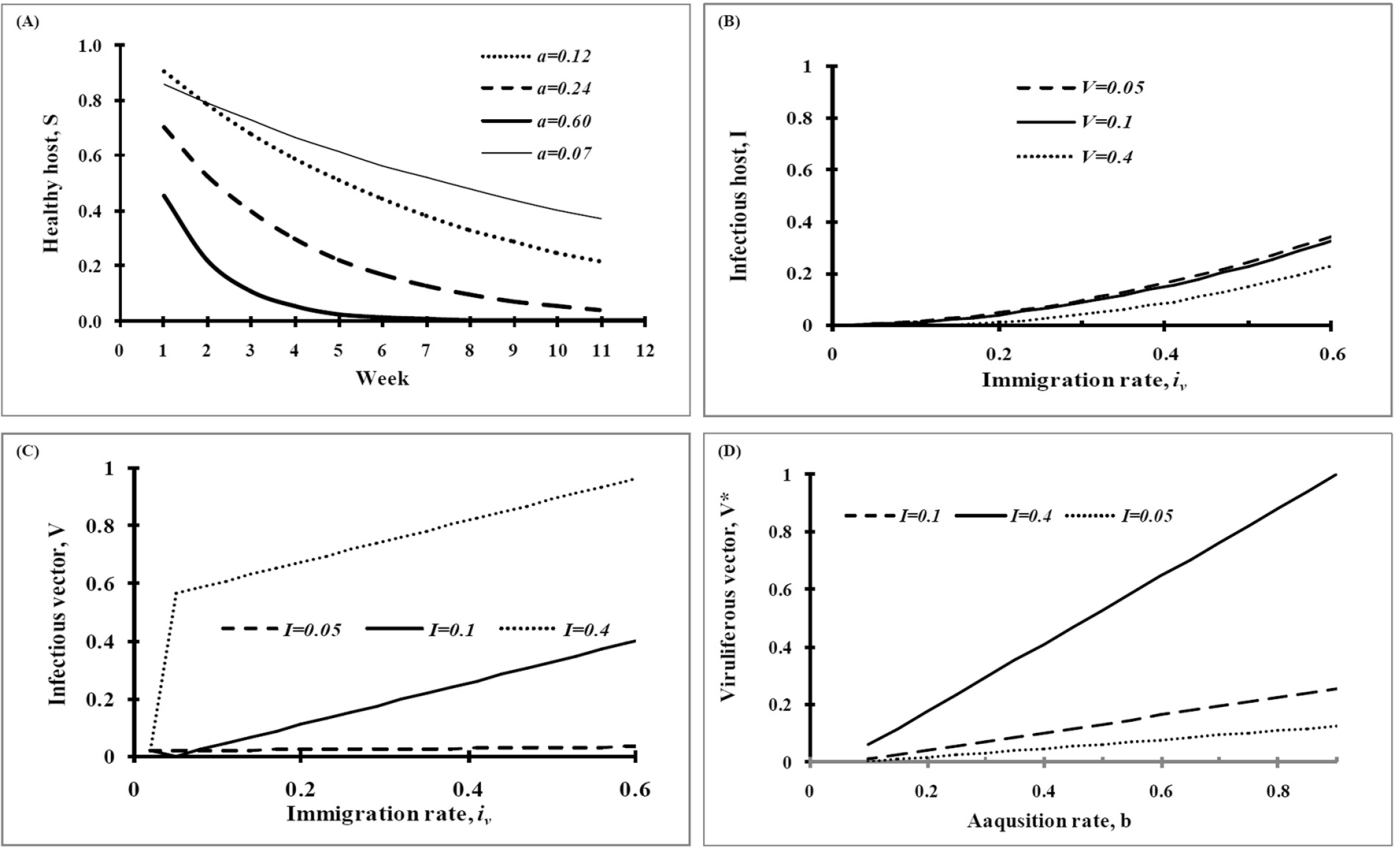
Parameter sensitivity on host and viruliferous whitefly population; A = transmission rate on healthy host; B = immigration rate on infectious host population; C = immigration rate on viruliferous vector and D = acquisition rate on viruliferous vector population.

Tripartite interaction captured in a dynamic model was used to assess the behavior of other parameters like immigration and emigration, death and birth rate of whiteflies which are difficult to observe but to be determined analytically. Sensitivity analysis was made to determine how changes in parameter values affected the epidemic process underpinning disease management clues. Changes in parameter values particularly for transmission rate (*a*), immigration rate (*i*_*v*_) and acquisition rate (*b*) were noted to have a marked influence on population of host as well as viruliferous vector. Transmission rate parameter (*a*) was observed to be responsive throughout the crop period indicating the importance of the vector activity in disease spread (Fig. 3A). Equilibrium population of infectious host, as well as viruliferous vector, was sensitive to immigration parameter (*i*_*v*_) as both the population got increased with the increase in parameter value. Increase in infectious host population was more at a lower level (0.05 and 0.1) than at a higher level (0.4) of vector population (Fig. 3B). It indicated immigration of viruliferous-vector is very important at the initial stage of virus transmission than at a later stage of epidemic development when high level of viruliferous population may be there. Measures to check migration of viruliferous vectors or avoiding migrant viruliferous vectors would be important in disease management. Immigration rate was also realized to be responsive for an increase in viruliferous vector population. The sudden jump in viruliferous vector population is likely to be in presence of an infectious host population (*I* = 0.4) even at a low rate of vector migration (Fig. 3C). Therefore, immigration of viruliferous vector was the most important parameter which acted concurrently in the flare-up of both the infectious population of host and vector. Acquisition rate was sensitive to increase viruliferous vector population at a higher level of infectious host (*I* = 0.4) as compared to the low level (*I* = 0.05–0.1) of population (Fig. 3D).

**Figure 4:**
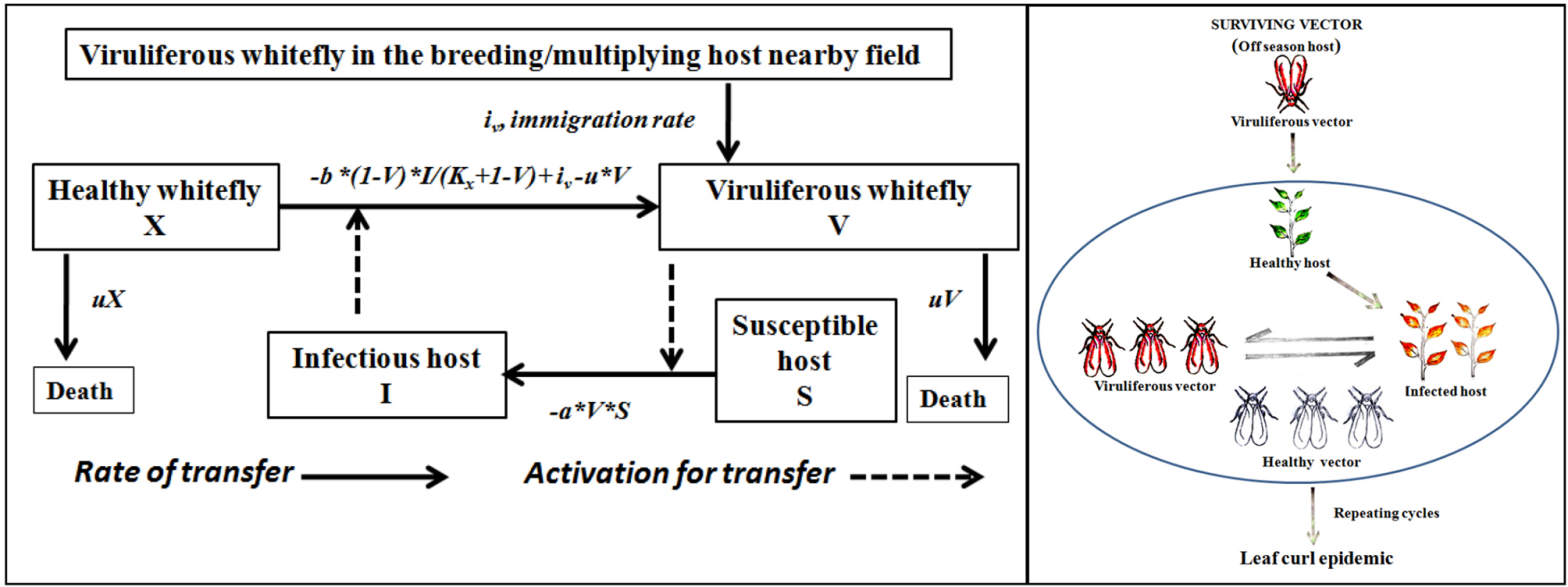
Schematic representation of dynamic tripartite interactions in leaf curl epidemic on a susceptible chili cultivar.  Left = interaction between host and vector leading to virus transmission; Right = migrant viruliferous vector infects host that serves as source of virus for aviruliferousvectors and which in turn infect healthy plants.

Therefore, tripartite interaction for leaf curl virus in chili was simplified through a schematic diagram maintaining the qualitative behavior of the process that is useful in understanding the dynamics of the transmission process and possible management interventions cues (Fig. 4).

Migrant viruliferous vector population appeared to play an important role in initial infection as well as to flare up both infectious host and virulifeorus vector population in the field for later exponential growth and make epidemic progress.

### Temperature influence on whitefly population abundance

**Figure 5:**
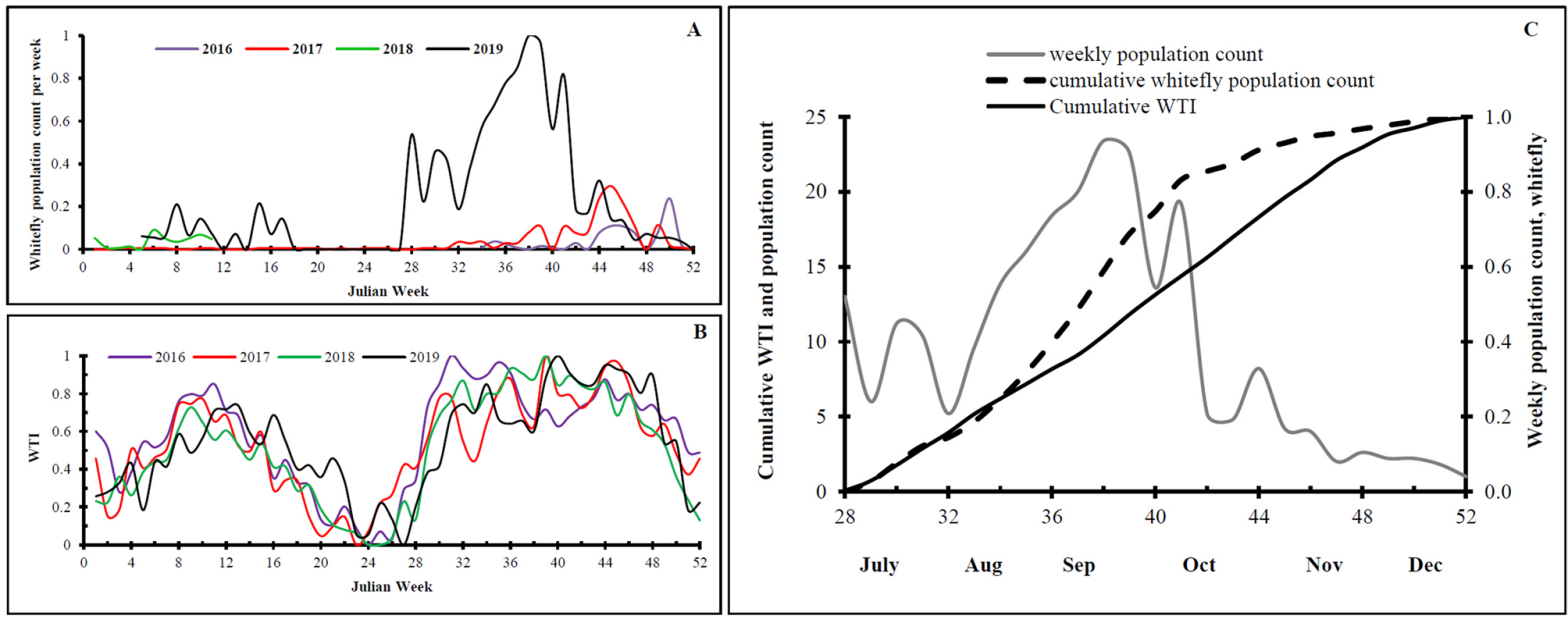
Temperature influence on whitefly population in experimental field IARI New Delhi, A = whitefly population count in Julian weeks; B = weekly temperature index in Julian weeks; and C = cumulative weekly temperature index and whitefly population count.

At the experimental site, whitefly population was observed in two distinct seasonal peaks, one during February–May and another in mid July–November period (Fig. 5A). Normalized population count was higher during July–November than February-May period. Temperature profile during Feb–March-mid April and mid July–November was within the threshold range (11–35 °C for whitefly population development) than the rest period. Whitefly developmental rate *r(T)* estimated based on threshold temperature (11–35 °C) and expressed as weekly temperature index [*WTI* = sum *r(T)*]was found to correspond with higher peak of whitefly population count in July–November (mean 22–35 °C) and low peak in February–May (10–32 °C) (Fig. 5B). Accumulated weekly temperature index and accumulated population count was in good agreement (r = 0.97). Therefore, accumulated weekly temperature index as an indicator of environmental suitability represented whitefly population (Fig. 5C).

It was evident that sum of developmental rate based on temperature profile reflected whitefly population abundance. Therefore, based on temperature index, whitefly population can be predicted for large areas as the geo-spatial temperature data is available.

### Prediction of spatio-temporal distribution of whitefly population abundance

**Figure 6:**
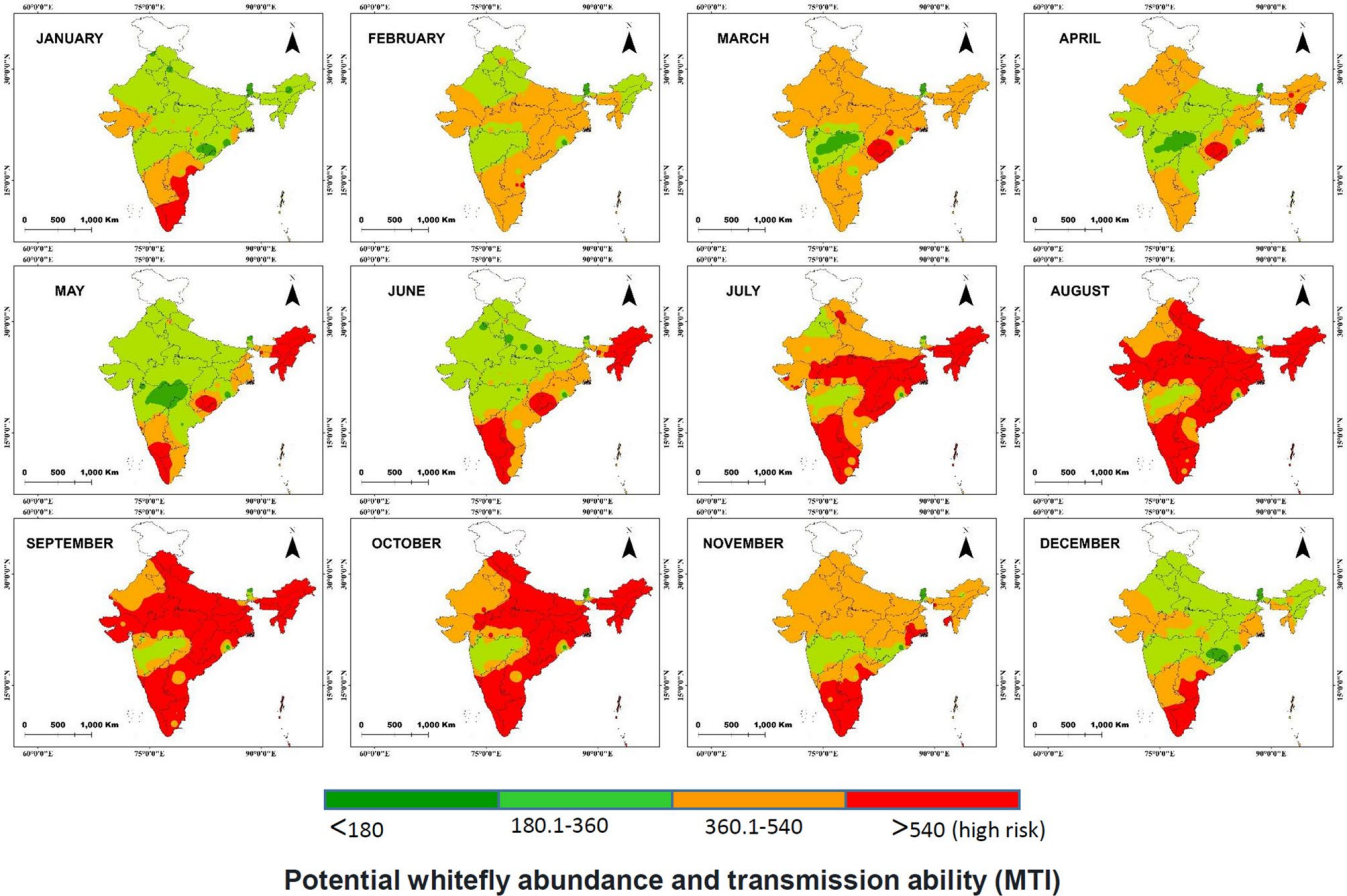
Prediction of spatio-temporal distribution of whitefly abundance (measured as growth and development index) in relation to monthly temperature index based on the prevalent temperature data (2001–2018) across the major chili growing areas in India; spatial interpolation (IDW in ArcGis 10.0 http://www.arcgis.com); red and dark green colors indicate high and low risk, respectively for whitefly population as well as leaf curl disease in chili.

Geographical distribution of monthly temperature index as an indicator of whitefly population abundance had shown distinct variation in intensity in time and space (Fig. 6). Monthly temperature index (*MTI*) or environmental suitability for whitefly abundance was at maximum intensity during July–November period almost throughout the country. In general, Southern peninsular region was observed to be suitable almost throughout the year as compared to other regions. It indicated major part of chili growing areas in the subcontinent found to be at high risk as possibility of vector population during this period is expected to be high. Marked increase in temperature index observed from July and continued till October. Chili planting during this season demands attention to check potential vector activity. During January–June period, Northwestern, Eastern and parts of Central India appeared to be relatively less favorable for whitefly development and likely to be less favorable for disease transmission.

Because of overlapping threshold temperatures of population abundance (11 °C, 22 °C and 35 °C) and transmission ability (measured at 15 °C, 25 °C and 35 °C) spatio-temporal distribution of vector abundance was appropriated as the measure of leaf curl risk. Therefore, spatio-temporal distribution pattern of vector abundance can be used as a reflection of probable disease risk or otherwise disease transmission ability.

Seasonal variation in leaf curl incidence in the major chili growing areas was confirmed based on preliminary survey and geographical distribution pattern for the disease reported^8,9^. In Northern India the chili crop grown during August-November period is affected by severe leaf curl epidemic, whereas the crop transplanted during December–January receives fewer incidences when whitefly population is comparatively low. Southern region is perpetually affected by severe epidemics as favorable temperature prevails almost throughout the year. It became evident that the period of high vector population and leaf curl incidence are overlapping as whitefly abundance and virus transmission are favored by similar temperature ranges.

## Discussion

Leaf curl disease dynamics has been simulated taking chili-ChiLCV-whitefly interaction into an epidemiological modeling frame work. Real process in tripartite interaction has been simplified based on mathematical models while maintaining the qualitative behavior of the mechanism useful in understanding the kinetics of transmission process. Immigration of viruliferous vectors and transmission parameters are the major parameters in the model noted to have a strong influence on the leaf curl epidemic. Otherwise, tripartite interaction is predominantly a vector mediation process. Current finding provides an important clue for emphasizing prevention of migrant vectors rather than the vector control normally thought for. Based on underlying principles of temperature influence on vector abundance and virus transmission spatio-temporal pattern of disease risk has been predicted. Spatio-temporal patterns for disease risk mapped across wide variants of agro-ecosystems may be used for crop planning if possible to avoid the period of high vector population. Modeling framework assumes susceptible chili cultivar and the viruliferous vectors are present in the agroecosystem. Therefore, parameter estimates are based on susceptible hosts. Adjustment of parameter values for host resistance may be required for general application of the finding. Epidemic analysis framework can be followed for testing and evolution of management strategies. A model framework of tripartite interaction necessary for evaluation of chili germplasms against leaf curl disease is now available.

For leaf curl dynamics in chili, *SI* model frame is good enough to explain epidemics as *E* (latently infected) and *R* (removed or post-infectious) categories are not essential elements in the model. Transmission studies have indicated inoculated plants 2-days onwards become PCR- positive. It appears virus multiplication in infected chili plants is quick and can serve as a potent source within a short period. Weekly observation is taken for estimation of infection proportion which covered enough multiplication time. Therefore, it may be the reason the model could explain dynamics without consideration of *E* type as latent period got covered in the observation interval. Based on the present finding it is surmised that latent period for leaf curl virus in chilli is shorter as far as transmission capability is concerned. Infected chili plants (maintained in isolation) are observed to remain infectious for more than 5 months as both acquisition and transmission of the virus is possible from those plants. Chili being a crop of 4–5-month duration, *SI* model serves the purpose for epidemic analysis without inclusion of *R* type as post-infectious category does not exist.

A high population of viruliferous vector as well as high disease incidence within a short period was an important feature in the epidemic. For such large number, both infectious host and vector are to be borne out of the field. It is possible under a situation where both the population goes increasing simultaneously as they are inter-dependent. Whitefly as persistent-circulative vector, requires virus acquisition from available infectious hosts in the field. Present finding suggests a gradual increase in both the population occurs in the field simultaneously. Basic reproduction number estimated has reflected a higher number of new infections in the next generation. Viruliferous migrant vectors make new infections or infectious hosts and new generation fly acquires virus from the newly infected host. Therefore, migrant viruliferous vector is the driving force to operate the tripartite interaction. We hypothesize a positive feedback mechanism is in work where few migratory viruliferous vectors produce few infectious hosts which in turn facilitates more viruliferous vectors and the process continues till restrictions are imposed in the agro-ecosystem. Positive feedback is a process in which the end products of action cause more of that action to occur in a feedback loop and leads to exponential growth^35,36^. Therefore, complete avoidance or intervention to prevent viruliferous vector population in the field is very important. As gradual built-up of both infectious host and vector happens at early stages of crop, complete prevention of migrant vectors at the start of chili planting is expected to keep the plants infection-free. Therefore, management of leaf curl is dependent on how best measures are taken to check migrant vector’s entry into the field without interference in intercultural operations. Prevention of infectious vector’s entry from the very beginning or complete avoidance of migrant vectors from the crop-start may be possible by covering the rows with synthetic plant covers that prevents insects but not interfering plant growth. Now-a-days plant covers are available. But the crop requires irrigation, fertilization and intercultural operations. Before going for management trials, there are important issues to resolve. Firstly, how long the plants are to be under complete cover as it hinders intercultural? It requires testing what is the minimum period of cover after which minimum tripartite interaction may happen but not affecting yield and quality. Secondly, what are the cares (say mulching to check weeds) at the time of transplanting to be taken so that plants are protected from vector and at the same time intercultural operations can be allowed without vector’s entry. Thirdly, the cost involvement for the alternatives to be used for vector prevention requires evaluation. Without sorting out these issues management trials are again poised to remain open without any conclusion.

Effect of vector aggregation affects transmission parameters^21^. Variation in transmission rate has been realized in the current study as the parameter estimates (*a*) with one whitefly was nine times higher than with ten flies (expected some degree of aggregation). Density of the host also affects vector mobility and therefore virus transmission^17^. Therefore, inclusion of aggregation parameter for both host and vector would have given a more precise simulation of the dynamics.

Both transmission and acquisition rates are affected by temperature and the unimodal response may possibly be a general phenomenon as far as vector transmissions of viruses are concerned. Similar behaviour of transmission is reported for aphid-transmitted viruses like Banana-bunchy-top in banana^27^ and leaf roll in potato^37^ where temperature thresholds (below 10 °C and above 35 °C) affected both the events. Maximum rate of transmission is obtained at 25 °C which is the normal temperature for aphid growth. For chili leaf curl, greater virus transmission ability (transmission and acquisition efficiency) at 25 °C and decreasing effect of temperature at or below 15 °C and at 35 °C or above, suggests vector population growth, as well as virus transmission, is remarkably influenced by temperature. Thermo tolerance and gene expression following heat stress in the whitefly *Bemisia tabaci* B and Q biotypes has shown low sensitivity in transmission for low or high temperature^38,39^. Temperature influence on virus transmission abilities and its approximation with vector abundance is important information that facilitated its spatio-temporal distribution. Geographical distribution of begomoviruses has been reported to be analogous to occurrence of whitefly in the world^40^. It is to be seen whether a similar pattern of temperature influence on the transmission is applicable for other plant viruses. If it is true, then prediction of vector abundance and transmission abilities may be used as a critical input for evolving management strategies against most virus diseases.

Seasonal whitefly population abundance has been predicted based on temperature thresholds for developmental rate. Potential vector growth as a map of potential risk of pest occurrence is an important component for management decisions^18^. Spatio-temporal distribution of whitefly abundance and virus transmission ability is approximated as leaf curl risk has shown good correspondence with empirical observation as well as available reports on leaf curl incidence^3,12^.

For the prediction of potential disease risk based on temperature thresholds, characteristic behavior of unimodal developmental response to temperature has been captured by three-parameter beta functions^41^. The beta function of three parameters is more robust to capture the developmental rate as parameters have meaningful biological interpretation than the two-parameter models^30,33,42–44^.

To sum up, leaf curl dynamics is established and migrant viruliferous vectors are denoted as the initiator of the epidemic process. For efficient management of the disease, emphasis must be on prevention of vector’s entry right from the stage of seedling preparation and transplanting. Temperature plays an important role, particularly in transmission process. Thus leaf curl risk prediction based on temperature may be a useful guide to evolve intervention strategies. The assessment framework may be also applicable to other viruses transmitted by whiteflies.

## Material and methods

### Population dynamical models for simulation of leaf curl epidemic

For population dynamics modeling, host population is divided into healthy (*S*, virus free), latent (*E*, carrying virus) and infectious (*I*, serves as virus source) and post-infectious or removed (*R*, no longer virus source) type. Similarly, whitefly population is divided into aviruliferous (*X*, virus free) and viruliferous (*V*, capable of transmission) type. Due to interaction, host population (*S*) is converted into latent category (*E*) once visited and probed by viruliferous whitefly (*V*) and after a period latent plants are transferred into infectious (*I*) type. Over-infectious or dead plants are called as post-infectious or removed (*R*) type.

For leaf curl dynamics, following population dynamics models previously used to explain tripartite interactions for plant viruses, were considered^21,23–26^.

1 $$dS/dt \, = \, {-} aVS$$

2 $$dI/dt = \, aVS$$

3 $$dX/dt \, = cX + cV \, {-} \, bXI/\left( { \, K_{x} + X} \right){-} uV \, + \, i_{x} {-} e_{x}$$

4 $$dV/dt \, = \, bXI/\left( { \, K_{x} + X} \right) \, + \, i_{v} {-} e_{v} {-} \, uV \, = \, b\left( {1{-} \, V} \right)I/\left( { \, K_{x} + 1{-} \, V} \right) \, + \, i_{v} {-} e_{v} {-} uV$$

For chili, host population was divided only into two categories, healthy *S*, and infectious *I* type. Chili plants once infected were observed to remain infectious till the end of the crop season. So post-infectious or removed *R* type was not included in the model. Similarly, vector population was categorized into aviruliferous *X*, and viruliferous *V* type. Since the vector was capable of virus transmission with 20 min inoculation feeding period, latent category was not included in the model.

In tripartite interaction, virus transmission between host and vector is dependent on parameters *a* (transmission or inoculation rate) and *b* (acquisition rate). Parameter *a* determines conversion or transfer rate of healthy host (*S*) into infectious category (*I*) which is dependent on the number of healthy host (*S*) and viruliferous vector population (*V*). Virus acquisition rate by an aviruliferous vector (X) is defined by parameter *b,* which is dependent on the number of healthy vector (*X*) and number of available infectious host (*I*). Other parameters included in the model are rate of immigration and emigration ofviruliferous (*i*_*v*_ and *e*_*v*_ respectively) and aviruliferous (*i*_*x*_ and *e*_*x*_ respectively) vectors, *c* and *u* birth and death rate respectively (both aviruliferous and viruliferous), Michaelis–Menten constant (*K*_*x*_).

Estimated parameters are tuned by fitting models on field data where infections were observed under natural conditions. Setting the model in a state of real situation (calibrated), behaviors of other parameters were assessed. Parameter sensitivity was performed on the equilibrium points of population of host and vector to see the impact of changes in their values make changes in host (S* and I*) and vector (V*) population.

Equilibrium points:

5 $$S* = 1 - I*$$

6 $$I* = \, \left( {u \, e_{v} + u^{2} V - u \, i_{v} } \right)/\left( {b\left( { \, i_{v} - \, e_{v} + \, i_{x} - \, e_{x} - uV} \right)} \right)$$

7 $$X* = \, \left( {e_{x} - i_{x} + V\left( {u - c} \right)} \right)/\left( {c - bI} \right)$$

8 $$V* = \, \left( {u \, e_{v} - u \, i_{v} - I\left( { \, i_{x} - \, e_{x} } \right) + I\left( { \, i_{v} - \, e_{v} } \right)} \right)/\left( {uI - u^{2} } \right)$$

The equilibrium points for host and vector population (S*, I* and V*) were derived from simpler equations of tripartite interaction to avoid quadratic roots in system solution.

Temperature influence on major transmission parameters was assessed for prediction of probable geographical distribution of the disease to explain epidemic process and predict disease risk.

### Parameter estimation-transmission (*a*) and acquisition (*b*) rates

Transmission or inoculation rate for the ChiLCV was estimated under semi-controlled conditions^26^. Ten viruliferous whiteflies were allowed to feed on eight chili plants (susceptible cv HPH-1041, Syngenta) grown in earthen pot (dai 10ʺ) and maintained in an insect proof cage. Experiment was performed under three levels of temperature exposure viz., 15 °C, 25 °C and 35 °C. Whiteflies were killed after 7 days by spraying insecticide (@1 ml/L Confidor, Bayer). Leaves from each plant were collected for virus detection through PCR (ChiLCV specific primer pairs) after 1, 2, 3, 4, 5, and 7 days’ post inoculation. Transmission rate (*a*) was estimated by fitting the dynamical model Eq. (1).

For estimation of acquisition rate (*b*), ten aviruliferous whiteflies were released on an infectious plant maintained in an insect proof cage. The experiment was performed at three temperatures viz., 15 °C, 25 °C and 35 °C to assess temperature influence on virus acquisition. Vectors were collected at 0, 10, 20 min, 1, 2, 4, 8, 12, 24, and 48 h interval for PCR detection (ChilCV specific primers). Acquisition rate, *b* was estimated fitting the dynamical model:

$$dV/dt \, = bXI = \, b\left( {1 - V} \right)I$$

*X* = number of aviruliferous vectors in time; *V* = number of virulifeorus vector in time; *I* = number of infectious plants in the exposure in time; *b* = acquisition rate, *X* encounters with *I.*

For fitting dynamic models, R software was used. Precision of the model was tested by comparing simulated values of healthy host and viruliferous vector population with observed values estimated through sampling in the field. The RMSE was used as a measure of the discrepancies between observed values and model simulations.

$$RMSE = \sqrt {\frac{{\sum\nolimits_{i = 1}^{n} {(x_{sim,i} - x_{obs,i} )^{2} } }}{n}}$$

where *x*_*sim,I*_ is the simulated value for healthy host and viruliferous vector, *x*_*obs,I*_ is the observed value for corresponding populations, and *n* is the number of observation, with a value of 12 in the present evaluation.

### Assessment of infectious host (*I*) and whitefly vector (*V*) populations in experimental field

In a field plot experiment at IARI New Delhi (18.6317°N, 77.2241°8E, 219.7 m), 500 seedlings of susceptible chili (cv HPH-1041) were transplanted in the second week of August 2019 and grown under normal package of practice without any management interventions.

For estimation of host (infectious) and vector population (viruliferous) samples of leaves and flies were taken from the first week of post transplanting. Weekly composite of 200 leaves, taking one leaf from the top of 200 randomly selected plants was prepared. Similarly, a composite of 100 whiteflies collected (using a suction device) from five randomly selected plants. Both leaf and whitefly samples were grouped for testing of ChiLCV through PCR. Group testing was done following the scheme mentioned in the Table S1. Uniform PCR protocol (ChiLCV specific primers) was followed for leaf and whitefly samples^45^ (Table S1). Proportion of infected leaf or whitefly (*P*) was estimated using the following formula^46–49^:

$$P = 1 - \left( {\left( {n - X} \right)/n} \right)^{1/m}$$

where *n* = number of groups made for leaf and whitefly samples; *m* = number of leaves or whiteflies in each group; and *X* = number of groups tested as PCR-positive.

At transplanting, leaf, as well as whitefly samples from the resident crops (cucurbits, brinjal and tomato) and border tree (*Leucana leucocephala*), were also collected for testing in similar methods of sampling.

Weekly proportion of infected leaves and flies based on PCR test was considered as the proportion of infectious host (*I*) and viruliferous vector (*V*) population in the field. Proportion of infected host and vectors estimated were considered as *I* and *V* as once they are infected remain infectious.

### Basic reproduction number (*R*_*0*_) for leaf curl in chili

Basic reproduction number calculated as the total number of infections arising from one newly infected host and whitefly introduced into the healthy host population^50^. It represents the ratio between subsequent generations at population level. The *R*_*0*_ was calculated as dominant *eigen* value of the next generation matrix (*A*) where the time step of the matrix is the generation time^51^:

$$A=\left(\begin{array}{cc}0& aS\\ bX& 0\end{array}\right)\left(\begin{array}{cc}\tau I& 0\\ 0& \tau Y\end{array}\right)$$

where *a* = transmission rate, *b* = acquisition rate, $\tau I$= time period an infected host remains infectious, $\tau Y$= time period the vector remains viruliferous, *S* and *X* = healthy host and non-viruliferous vector population density (number/m^2^), respectively. *R*_*0*_value calculated from the equation^50,52^:

$$R_{0}^{2} - \alpha S\tau Y \cdot bX\tau I = 0$$

### Prediction of whitefly population abundance based on temperature influence

Whitefly population was observed during 2016–2019 at IARI New Delhi experimental field where regular planting of chili was followed. Flies from five plants were collected through a suction device at the weekly intervals (Julian week) and the mean count was expressed as a normalized population per week^53^.

$${\text{Normalized}}\;{\text{population}}\;{\text{count }} = \left( {Mean \, count - Min_{mean \, count} } \right)/ \, (Max_{mean \, count} - \, Min_{mean \, count} )$$

To assess the effect of temperature on developmental rate [r*(T)*] for whitefly population, non-linear beta function which utilizes cardinal temperatures of whitefly growth and development was used^41,54^:

$$\begin{gathered} {\text{r}}(T) = \left[ {\left( {T_{{upper}} - T_{{air {\text{-}} h}} } \right)/\left( {T_{{upper}} - T_{{opt}} } \right)} \right]*\left[ {{{\left( {T_{{air {\text{-}} h}} - T_{{lower}} } \right)} \mathord{\left/ {\vphantom {{\left( {T_{{air {\text{-}} h}} - T_{{lower}} } \right)} {\left( {T_{{opt}} - T_{{lower}} } \right)}}} \right. \kern-\nulldelimiterspace} {\left( {T_{{opt}} - T_{{lower}} } \right)}}} \right]\wedge {{\left( {T_{{opt}} - T_{{lower}} } \right)} \mathord{\left/ {\vphantom {{\left( {T_{{opt}} - T_{{lower}} } \right)} {\left( {T_{{upper}} - T_{{opt}} } \right)}}} \right. \kern-\nulldelimiterspace} {\left( {T_{{upper}} - T_{{opt}} } \right)}} \hfill \\ {\text{if}}\;\;T_{{lower}} \le T \le T_{{upper}} \;\;{\text{and}}\;\;0\;\;{\text{otherwise}} \hfill \\ \end{gathered}$$

where, *T*_*upper*_ (35 °C),* T*_*lower*_ (11 °C) and *T*_*opt*_ (22 °C) are the upper, lower and optimum threshold temperatures, respectively, for egg laying and adult development^4,31,55,56^. *T*_*air-h*_ is the hourly air temperature (°C).

Daily temperature for the year 2016–2019 was collected from the Institute’s Meteorological observatory IARI New Delhi (28.7041° N, 77.1025° E). Daily minimum (*T*_*min*_) and maximum (*T*_*max*_) temperatures were converted into hourly temperatures (*T*_*air-h*_) based on the standard formula^57^, which provides smooth transformation from minimum to maximum air daily temperature:

$$T_{air - h} = \left( {T_{max} + T_{min} } \right)/2 + \left( {T_{max} - T_{min} } \right)/2*cos(0.2618*(h - TimeVar)$$

where *TimeVar* is the hour of the day corresponding to the time of occurrence of *T*_*max*_.

For estimation of temperature influence, hourly temperature (*T*_*air-h*_) was corrected for upper and lower threshold limits for whitefly growth and development. Hourly estimates of *r (T)*were calculated and daily *r(T)* were obtained summing hourly values for the day. Weekly temperature index (*WTI*) was calculated by summing daily *r(T)* values for the week:

$$WTI={\sum }_{t1}^{t2}r\left(T\right)$$

where *t1* and *t2* are the r *(T)* for the first and last day of the week.

Accumulated temperature index (*WTI*) for the crop period (August–November) was related to the accumulated population of whitefly count as a measure of environmental suitability for population abundance.

### Prediction of spatio-temporal distribution for whitefly population abundance

For prediction of whitefly population abundance based on environmental suitability, daily minimum and maximum temperature were downloaded from the IMD portal (http://mausam.imd.gov.in). Temperature data for the period 2001–2018 from 85 geo-referenced meteorological stations of important chili growing states of India was considered. For probable distribution of vector population, monthly cumulative temperature index (*MTI*) was estimated.

$$MTI={\sum }_{t1}^{t2}r\left(T\right)$$

where *t1* and *t2* are the r *(T)* for the first and last day of the month.

The cumulative temperature indices (*MTI*) were plotted using *ArcGis10.0* (http://www.arcgis.com). For continuous surface, IDW (inverse distance weightage) interpolation technique was applied and spatial maps were generated. For verification of predicted distribution, a preliminary survey across major chili growing areas conducted for leaf curl incidence.

## Supplementary Information

Supplementary Information.
