# Supplementary material for: Simulation of leaf curl disease dynamics in chili for strategic management options
Source: Sci Rep. 2021 Jan 13;11:1010. doi: 10.1038/s41598-020-79937-0 (PMC7806845; doi:10.1038/s41598-020-79937-0)
Supplement: Supplementary file 1 — Supplementary Information. [file 41598_2020_79937_MOESM1_ESM.docx]

**Simulation of leaf curl disease dynamics in chili for strategic management options**

Buddhadeb Roy, Shailja Dubey, Amalendu Ghosh, ShaluMisra Shukla, Bikash Mandal &Parimal Sinha*

**Supplementary Table**

**Table S1** Sampling procedure for estimation of proportion of infection in leaf and whitefly population through PCR detection of ChiLCV.

| Step | Composite sampling and PCR detection for ChiLCV | |
| --- | --- | --- |
|  | Leaf | Whitefly (Wf) |
| 1 | 200 leaves collected from 200 individual plants | 100 Wf collected from five plants |
| 2 | Divided into two groups of 125 and 75 leaves.   - First, 125 leaves again divided into 5 groups consists of 25 leaves. - Remaining 75 leaves divided into 3 groups consists of 25 leaves in each group. | Divided into two group of 70 and 30 Wf.   - First, 70 Wf divided into 7 groups consists of 10 Wf. - Remaining 30 Wf divided into 3 groups consists of 10 flies in each group. |
|  | PCR detection using specific primers pairs | |
|  | If not all PCR-positive (in first 5 groups) then follow PCR detection for the remaining 3 group | |
|  | Calculate *P** *= 1-((n-X)/n)^1/m^; n*= number of groups; *m*= number of leaves or whiteflies in each group; *X*= number of groups tested PCR- positive. | |
|  | n= (5+3)=8  m=25 | n=7+3=10  m=10 |
| 3 | If all 5 groups PCR-positive in step 2 then remaining 75 leaves divided in to two groups of 50 and 25 leaves. | If all 5 groups PCR-positive in step 3 then remaining 30 WF divided in to two groups of 20 and 10 WF. |
|  | Group of 50 divided into 10 groups each group having 5 samples. | Group of 20 divided into 5 groups each group having 4 samples. |
|  | PCR detection using specific primers pairs | |
|  | If not all PCR-positive (in 10 groups) then calculate P* with n=10 and m=5 | If not all PCR-positive (in 5 groups) then calculate P* with n=5 and m=4 |
| 4 | If all 10 groups PCR-positive in step 3 then for the remaining 25 leaves PCR detection for each leaf. | If all 5 groups PCR-positive in step 3 then for remaining 10 WF, PCR detection with single fly. |
|  | Calculate P* as simple proportion out of 25 leaves | Calculate P* as simple proportion out of 10 flies |
| PCR METHOD:  For ChiLCV detection, genomic DNA was isolated from the leaf and whitefly samples (CTAB method). PCR was performed in 25 µl reaction mixture containing 20-30 ng genomic DNA, 2.5µl of 10X PCR buffer (Thermo Scientific), 1 µl of each forward (BMf-861) and reverse (BMr-862) primers, 260 µM of dNTP (Thermo Scientific) and 2U of DreamTaq polymerase (Thermo Scientific).  Specific pair primers (BMf-861:5’GAGTCTAGACACGATGTAG-3’; BMr-862: 5’CATCAGAGCATTCTCACT-3’) was used to amplify approximately 453 bp sequence partially overlapping the putative AV1, AC3 and AC2 genes (Mandal, 2017) following amplification steps: 94^o^C for 3 m, 34 cycles of 94^o^C for 30 s, 52^o^C for 45 s and 72^o^C for 30s; 72^o^C for 10 m (Thermocycler, Hi-Media). PCR-positive of a sample was confirmed by the presence of specific (760 bp) band in agarose gel (2%). | | |
